# Hemp (*Cannabis sativa* L.) Phytochemicals and Their Potential in Agrochemical, Cosmetic, and Food Industries: A Review

**DOI:** 10.3390/ijms27031146

**Published:** 2026-01-23

**Authors:** Daniela Trono

**Affiliations:** Research Centre for Cereal and Industrial Crops, Council for Agricultural Research and Economics (CREA), S.S. 673, Meters 25200, 71122 Foggia, Italy; daniela.trono@crea.gov.it

**Keywords:** hemp, phytochemicals, biopesticides, cosmetics, foods, dietary supplements

## Abstract

Hemp is a high-yield crop traditionally cultivated for fiber used in products such as paper, textiles, ropes, and animal bedding, and more recently for sustainable applications in biofuels, insulation, and bioplastics. Beyond fiber, hemp is rich in phytochemicals. More than 500 compounds including cannabinoids, terpenes, phenolics, phytosterols, and tocopherols are accumulated in leaves, flowers, and seeds, which are typically considered waste products in the fiber industry. These compounds exhibit antioxidant, anti-inflammatory, neuroprotective, and antimicrobial properties, which have stimulated research into their pharmaceutical potential. However, hemp phytochemicals also find applications in other industrial sectors, including agrochemistry as natural insecticides, cosmetics for skin and hair care, and food and dietary supplements due to their associated health benefits. In light of this, the present review aims to give an overview of the available literature on the most common applications of hemp tissues, hemp extract, and purified hemp phytochemicals in agrochemical, cosmetic, and food sectors. This will be helpful to critically assess the current state of knowledge in this field and contribute to the ongoing debate over the natural and sustainable applications of hemp by-products.

## 1. Introduction

*Cannabis sativa* is an annual herbaceous plant from the Cannabaceae family that was first domesticated in early Neolithic times in East Asia and subsequently spread to other parts of the globe [1]. *C. sativa* is classified into two categories based on its intended use: marijuana and hemp. Because of its high levels of Δ^9^-tetrahydrocannabinol (THC), which are sufficient to produce psychoactivity, marijuana is mostly used for recreational and medicinal purposes. By contrast, hemp contains less than 0.3% THC, an amount insufficient to cause psychoactivity, and high levels of cannabidiol (CBD) or cannabigerol (CBG) [2].

Hemp has been grown primarily for its fiber, which has been used for thousands of years for a variety of purposes, including clothing and shoes, ropes and cords, and papermaking; also, hemp seeds have been used as a food ingredient, and leaves as compost, mulch, and animal bedding [3]. However, in the early 20th century, the competition from other natural fibers, such as cotton and jute for textile applications, as well as the rapid development of synthetic fibers, has led to a decline in hemp cultivation, which came to a complete stop in the mid-20th century, when it was made illegal due to its close botanical relationship with marijuana [3]. In the 21st century hemp has been re-established as a legal crop, and its cultivation has resumed rapidly, thus paving the way for new environmentally friendly applications, which include insulation, bioplastics, and biofuels [4].

Other recently explored industrial applications of hemp are linked to the rich phytochemical profile of this crop. The *C. sativa* plant produces a unique class of terpenophenolic compounds, called cannabinoids, as well as non-cannabinoid compounds mainly represented by terpenes and phenolic compounds [5]. Although the therapeutic properties of cannabinoids have been extensively studied and their pharmaceutical applications have exploded over the last years, the other compounds have no reason to envy them, as they have also been associated with potent health-promoting properties, including antioxidant, anti-inflammatory, antimicrobial, and neuroprotective effects. All these compounds can also act synergistically, giving rise to the so-called “entourage effect”, which generate a greater therapeutic effect than a single compound could achieve alone [6].

However, beyond their potential pharmaceutical applications, hemp phytochemicals are increasingly recognized for their use in agrochemical, cosmetic, and food industries. Indeed, thanks to their insecticidal properties, hemp phytochemicals are successfully employed in the production of natural pesticides [7]. For their antiaging, antioxidant and moisturizing effects, they are used as ingredients in skincare and haircare products [8]. Additionally, hemp phytochemicals add nutraceutical value and potential health benefits to a range of food products and dietary supplements that contain hemp and its derivatives as ingredients [9].

In light of the above, it can be stated that, thanks to their phytochemicals, the industrial applications of hemp leaves, inflorescences, and seeds, which are typically considered waste products in the fiber industry, may represent a complementary strategy to improve hemp competitiveness in comparison to other crops and may be of interest in a circular economy perspective, in which waste material is exploited to obtain industrially useful products. In this context, the present review aims to provide an overview of the hemp phytochemicals of industrial interest and explore the current state and future directions of their potential applications in the agrochemical, cosmetic, and food sectors.

## 2. Phytochemical Profile

**Figure 1:**
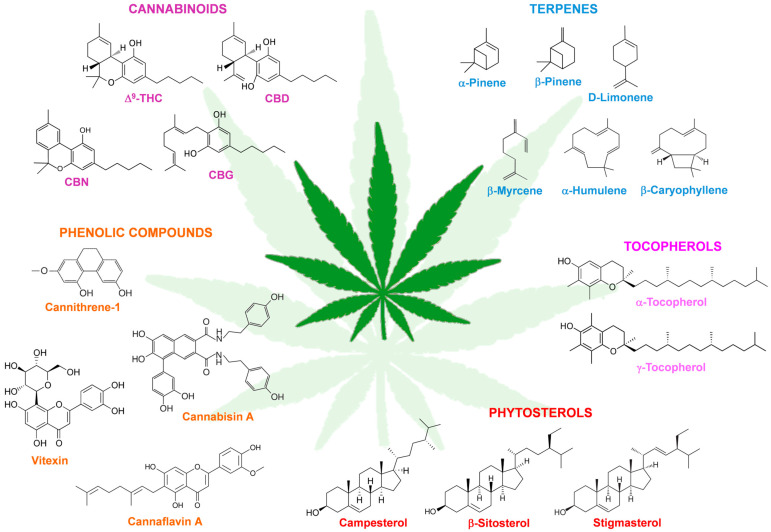
Examples of the most abundant phytochemical compounds identified in the *C. sativa* plant. CBD, cannabidiol; CBG, cannabigerol; CBN, cannabinol; Δ^9^-THC, Δ^9^-tetrahydrocannabinol.

More than 500 phytochemical compounds have been identified in *C. sativa*, including 125 cannabinoids, 120 terpenes, 42 phenols, and 34 flavonoids [5]. Tocopherols and phytosterols are also present, albeit in lower amounts. Below, and in Figure 1, is an overview of the major classes of hemp phytochemicals. A detailed treatment of this topic is beyond the scope of this review, and readers are referred to excellent reviews that provide in-depth descriptions of the chemical structures, isolation methods, biosynthesis, and properties of the major phytochemicals identified in *C. sativa* [5,10,11].

### 2.1. Cannabinoids

Cannabinoids are a group of phytochemicals that, together with terpenes, are synthesized and accumulated in the glandular trichomes that coat the inflorescences and most of the aerial parts of the *C. sativa* plant. Low amounts of cannabinoids have also been detected in the roots, whereas their presence in the seeds is the result of contamination during the harvesting process, when the seeds can come into contact with the inflorescences and leaves [12]. The most abundant cannabinoids found in *C. sativa* plants are THC, CBD, CBG and cannabinol (CBN) [10] (Figure 1). Female inflorescences are the primary source of cannabinoids, making them the ideal starting material for extracting these compounds for use in cannabinoid-based products [13].

The biosynthesis of cannabinoids begins with the production of cannabigerolic acid (CBGA) from the combination of geranyl pyrophosphate with olivetolic acid, a reaction catalyzed by the geranyl-pyrophosphate—olivetolic acid geranyltransferase. CBGA then serves as substrate for specific oxidocyclases, which convert CBGA into the cannabinoid acids Δ^9^-tetrahydrocannabinolic acid (THCA), cannabidiolic acid (CBDA), and cannabichromenic acid (CBCA). These acidic cannabinoids can be non-enzymatically decarboxylated to their neutral forms when exposed to light or heat [10].

Of all the cannabinoids, THC and CBD are those responsible for many of the therapeutic effects associated with *C. sativa*. Most of the biological properties of cannabinoids are mediated through the human endocannabinoid system (ECS), a complex network of receptors (CB1 and CB2) and endogenous ligands that play a vital role in regulating various physiological functions. THC and CBD extracted from *C. sativa* plants, as well as synthetic THC analogues, are currently used to produce drugs for the treatment of nausea, chronic pain, and seizures, but they have also shown promise in treating neurodegenerative diseases, anxiety, post-traumatic stress disorder, various types of cancer, and glaucoma [14]. Recent studies have highlighted therapeutic potential also for CBG, especially in treating neurologic disorders and inflammatory bowel disease [15].

### 2.2. Terpenes

Terpenes are the second most abundant class of phytochemicals in *C. sativa* and are responsible for its distinctive odor and flavor. They are mainly represented by monoterpenes and sesquiterpenes, produced in abundance in the trichomes of the inflorescences and the leaves. α-Pinene, β-pinene, D-limonene, and β-myrcene are the monoterpenes detected at the highest levels, whereas β-caryophyllene and α-humulene are the most abundant sesquiterpenes [5] (Figure 1). Carotenoids, mainly β-carotene, lutein, and zeaxanthin, have also been detected in the inflorescences [16,17] and the seeds [18], whereas the triterpenes friedelin and epifriedelanol have been found in the roots [19].

Terpenes are synthesized through the cytosolic mevalonate pathway and the plastidial methylerythritol phosphate pathway. These pathways generate the isoprene units (C_5_H_8_) that are joined together in a head-to-tail condensation by specific terpene synthases. Depending on the number of linked isoprene units, the resulting terpenes are monoterpenes (C10), sesquiterpenes (C15), diterpenes (C20), sesterterpenes (C25), triterpenes (C30), sesquarterpenes (C35), tetraterpenes (C40), and polyterpenes (>C40) [20].

Terpenes found in *C. sativa* present a wide array of pharmacological properties that include but are not limited to antioxidant, anti-inflammatory, antidiabetic, analgesic, anticonvulsive, antidepressant, anxiolytic, anticancer, neuroprotective, antiallergic, and antibiotic effects [21]. Also, many terpenes found in *C. sativa* show promise as natural insecticides, as they are able to inhibit the acetylcholinesterase activity in insects, a key enzyme in nerve function, causing insects to die [22].

### 2.3. Phenolic Compounds

The most abundant phenolic compounds identified in *C. sativa* belong to the classes of flavonoids, stilbenes, and lignans [11]. Flavonoids are mainly represented by vitexin, isovitexin, orientin, quercetin, apigenin, luteolin, and kaempferol in their methylated, glycosylated, prenylated, or geranylated forms, and by cannflavins, which are methylated isoprenoid flavones uniquely isolated from *C. sativa*; stilbenes belong to three structural types, namely, phenanthrenes, dihydrostilbenes and spiroindans, which include cannithrene-1 and -2, denbinobin, canniprene, dihydroresveratrol, cannabispiran, and cannabispirenone A and B, while the most represented lignans are amides and lignanamides, the latter including cannabisin-like compounds (of the types A-, B-, C-, D-, E-, F-, and G) accumulated in abundance in seeds [11] (Figure 1).

Phenolic compounds are produced through the phenylpropanoid pathway, which starts with the conversion of phenylalanine to cinnamic acid catalyzed by the phenylalanine ammonia-lyase. Subsequent reactions convert cinnamic acid to *p*-coumaroyl CoA, which serves as precursor for the biosynthesis of the different classes of phenolic compounds [23].

Phenolic compounds provide a wide range of health benefits linked to their ability to neutralize free radicals, thereby reducing the risk of chronic diseases associated with oxidative stress, such as cancers, cardiovascular, and neurodegenerative diseases [10]. Also, cannflavins have potent anti-inflammatory properties thanks to their ability to inhibit the prostaglandin E_2_ synthase and the 5-lipoxygenase [24]. Anti-inflammatory, anti-cancer, antiviral, and antiparasitic effects have been attributed to various stilbenes [25], while cannabisins B isolated from hemp seeds have shown antiproliferative activity in hepatoblastoma cells [26].

### 2.4. Other Phytochemicals

Although in smaller amounts, tocopherols and phytosterols have also been detected in the tissues of *C. sativa* plant. Tocopherols are among the most important lipid-soluble antioxidants able to quench free radicals in cell membranes, thereby preventing lipid oxidation. In *C. sativa*, tocopherols are mostly found in seeds, with γ-tocopherol being the predominant isomer [18], but their existence has also been documented in inflorescences, where α-tocopherol prevails [27] (Figure 1). Potential health benefits of tocopherols include the prevention of certain types of cancer, cardiovascular disease, and other chronic diseases, such as diabetes and obesity [28].

Phytosterols are cholesterol-like molecules found in all plants, with the highest levels detected in vegetable oil seeds. Consistently, hemp seeds are a good source of phytosterols, with β-sitosterol being the most abundant followed by campesterol, Δ_5_-avenasterol, and stigmasterol [29] (Figure 1). Evidence exists that phytosterols alleviates some metabolic disorders such as hypercholesterolemia, diabetes, obesity, and hypertension. Their most essential function is to decrease cholesterol absorption, leading to dramatic reduction in total cholesterol and low-density lipoprotein cholesterol levels [30].

## 3. Biopesticides

One of the main roles of phytochemicals in plants is to offer a variety of defenses against insects, parasites, and predators [31]. For this reason, plant-based pesticides are emerging as safe, efficient, and environmentally friendly substitutes for chemical pesticides in the fight against plant diseases and arthropods that transmit diseases to humans and animals. As for hemp, several studies have revealed that aqueous and solvent extracts, as well as essential oils (EOs) and purified phytochemicals from leaves or inflorescences effectively repel and/or inhibit the growth of insects and plant microbial pathogens [7].

### 3.1. Biopesticides Based on Purified Hemp Phytochemicals

Regarding purified hemp phytochemicals, studies have exclusively focused on cannabinoids and most of them have tested the effect of these compounds on insects harmful to plants. The first study dates back to the 1980s when Rothschild and Fairbairn [32] sprayed the surface of cabbage leaves with 1% CBD or 1% THC and observed that the cabbage butterfly (*Pieris brassicae*) laid 2–3 times less eggs on leaves sprayed with THC than on those sprayed with CBD (Table 1). The insecticidal and/or antifeedant activity of cannabinoids against herbivorous insects has also been highlighted by recent studies. The feeding study carried out by Park and coworkers [33] revealed that the addition of 2 mM CBD to the diet of the tobacco hornworm (*Manduca sexta*), a common pest of plants in the Solanaceae family, inhibited the growth and development of the larvae and increased their mortality (Table 1). Similarly, Stack and coworkers [34] observed that the addition of CBDA or CBGA at concentrations ranging from 0.1% to 1.0% to the diet of the cabbage looper (*Trichoplusia ni*), a serious pest of cruciferous plants, decreased the growth and survival of the larvae, up to reach 100% mortality at the highest concentration, whereas Abendroth and coworkers [35] observed that the larvae of the fall armyworm (*Spodoptera frugiperda*), a pest that damages a wide variety of crops, showed a dose-dependent decrease in growth and consumption when fed CBD concentrations ranging from 5% to 15% (Table 1).

CBD also showed pesticidal effect against stored product pests. A dose-dependent larvicidal activity was reported for 3% CBD oil against larvae of flou beetle (*Tribolium confusum*), saw-toothed grain beetle (*Oryzaephilus surinamensis*), and meal moth (*Plodia interpunctella*), which infest wheat, rice and corn seeds, with a mortality of up to 100% for the first two and 76% for the third one at the highest dose tested (90 mg mL^−1^) [36] (Table 1).

**Table 1:** Representative studies on the efficacy of purified cannabinoids as biopesticides.

Treatment	Target Organism	Effect	Ref.
*Insects harmful to plants*			
1% CBD or 1% THC sprayed on cabbage leaves	Cabbage butterfly (*Pieris brassicae*)	2–3 times less eggs deposited on cabbage leaves sprayed with 1% THC than on those sprayed with 1% CBD	[32]
0.01, 0.1, 1 mM, and 2 mM CBD incorporated into the artificial diet	Tobacco hornworm (*Manduca sexta*)	No effects at CBD ≤ 1 mM 20% smaller size, 2.2-times lower weight, and 60% lower larval survival rate at 2 mM CBD	[33]
0.001%, 0.01%, 0.1%, and 1.0% CBDA or CBGA painted on the surface or incorporated into the artificial diet	Cabbage looper (*Trichoplusia ni*)	No effects at CBDA and CBGA < 0.1% Decreased larval survival and growth at CBDA and CBGA ≥ 0.1% and 100% larval mortality at 1.0% CBDA	[34]
5%, 10%, and 15% CBD incorporated into the artificial diet	Fall armyworm (*Spodoptera frugiperda*)	Dose-dependent inhibition of consumption and growth No effect on digestibility and conversion efficiency	[35]
*Insects harmful to stored products*			
15, 45, and 90 mg mL^−1^ CBD oil (3%) sprayed on wheat, corn, and rice seeds	Flou beetle (*Tribolium confusum*) Saw-toothed grain beetle (*Oryzaephilus surinamensis*) Meal moth (*Plodia interpunctella*)	17–100%, 17–93%, and 26–83% larval mortality for *T. confusum* on wheat, corn, and rice seeds, respectively 17–100%, 36–96%, and 67–100% larval mortality for *O. surinamensis* on wheat, corn, and rice seeds, respectively 16–76%, 13–60%, and 33–63% larval mortality for *P. interpunctella* on wheat, corn, and rice seeds, respectively	[36]

CBD, cannabidiol; CBDA, cannabidiolic acid; CBGA, cannabigerolic acid; THC, Δ^9^-tetrahydrocannabinol.

To date, it is unclear how exactly CBD affects insects, which are known to lack the canonical cannabinoid CB1 and CB2 receptors [7]. A number of studies have reported the ability of CBD to modulate different biochemical targets in insects, likely through the interaction with other G-protein-coupled receptors. For instance, the reduced growth of *S. frigipeda* larvae fed increasing doses of CBD was found to be accompanied by a decrease in protease and cytochrome P450 activities, which might limit the detoxification response of the larvae by lowering the availability of amino acids required for the biosynthesis of the detoxifying enzymes [35]. Further, CBD-fed *M. sexta* larvae presented altered signal transduction of the ventral ganglion [33], and down-regulation of genes involved in exoskeleton development, such as cuticle-like and endochitinase, which led to a decrease in the amount of cuticle deposited [37].

### 3.2. Biopesticides Based on Hemp Essential Oil

Numerous studies have highlighted a toxic effect of EOs isolated from different hemp cultivars and tissues on insects and other organisms harmful to humans. Wanas and coworkers [38] tested the insecticidal activity of an EO obtained from hemp inflorescences against the yellow fever mosquito (*Aedes aegypti*) and observed that the EO had a biting deterrent activity comparable to that observed for *N*,*N*-diethyl-meta-toluamide, one of the most effective insect repellents [39], along with a low LC_90_ (90% lethal concentration) value (38.7–38.9 ppm) (Table 2). Interestingly, the authors also found that hemp EO had a significantly greater insecticidal activity than EO from a chemotype II cultivar (LC_90_ of 40.6–42.6 ppm), whereas EO from a chemotype I cultivar was ineffective. Additionally, EOs derived from the inflorescences of three distinct cultivars, Futura 75, Felina 32, and Kompolti, were found to be effective, albeit to varying degrees, against southern house mosquito (*Culex quinquefasciatus*) larvae (LC_90_ ranging from 142.3 μL L^−1^ to 700.9 μL L^−1^) and housefly (*Musca domestica*) adults (LD_90_ ranging from 212.9 μg adult^−1^ and 213.5 μg adult^−1^) [40,41,42] (Table 2). The EO derived from the leaves of cultivar Futura 75 was also found to be effective against these two insects, but less so than the EO extracted from the inflorescences of the same cultivar (LC_90_ of 410.3 μL L^−1^ vs. 199.1 μL L^−1^ for mosquito larvae and LD_90_ of 428.7 μg adults^−1^ vs. 212.9 μg adults^−1^) [40].

Other mosquito species highly sensitive to hemp EOs were the malaria vectors *Anopheles stephensi* and *Anopheles gambiae* that, when exposed to 100 ppm EOs from the inflorescences of the monoecious cultivar Felina 32 or from the male and female inflorescences of the dioecious cultivar Carmagnola Selezionata, showed 82.7–91.6% and 79.6–100% mortality of larvae and pupae, respectively [43] (Table 2). Albeit with low efficacy (LC_90_ of 693.999 μL L^−1^), a commercial EO was found to be effective against the larvae of the Asian tiger mosquito (*Aedes albopictus*) that can transmit several diseases including dengue, chikungunya and Zika [44] (Table 2). The same EO was successfully tested against the freshwater snail (*Physella acuta*), an intermediate host for nematodes and trematodes that cause diseases in animals and humans, achieving 100% mortality at a concentration of only 100 μL L^−1^ [44] (Table 2).

Hemp EOs were also tested against ectoparasites of veterinary importance. The same EO from cultivar Felina 32 inflorescences successfully used against housefly and southern house mosquito [41] was found to be effective against the poultry red mite (*Dermanyssus gallinae*) that infests poultry and other birds, and the camel tick (*Hyalomma dromedarii*) that infests camel and cattle, with a LC_90_ of 493 μg mL^−1^ and 517 μg mL^−1^, respectively [45]. A hemp EO containing γ-elemene and caryophyllene oxide as its major components (16.2% and 14.2%, respectively) and a very low amount of monoterpenes showed its toxicity against the cat flea (*Ctenocephalides felis felis*), a vector of several pathogenic agents to animals and humans, inducing 100% mortality of eggs, larvae and pupae at 200, 400 and 1600 μg cm^−2^, respectively, and 90% adult mortality at 2000 μg cm^−2^ [46] (Table 2). An EO extracted from hemp leaves also showed a dose-dependent acaricidal activity against the mite *Varroa destructor*, an ectoparasite that attacks honeybees, reaching 95.4% mortality at a concentration of 15% [47] (Table 2). The effectiveness of the hemp EO was found to be higher compared to EOs from sage (*Salvia officinalis*) and laurel (*Laurus nobilis*) that at the same concentration showed an acaricidal activity of 81.08% and 68.96%, respectively.

Hemp EOs were also found to be effective against plant pests. The EOs obtained from the inflorescences and leaves of the cultivar Futura 75 and the inflorescences of the cultivar Felina 32, successfully used against the southern house mosquito and the housefly, were found to be effective also against the tobacco cutworm (*Spodoptera littoralis*), with the EO obtained from the inflorescences of the cultivar Futura 75 showing the highest toxicity (LC_90_ of 89.3 μL L^−1^ vs. 221.5–313.1 μL L^−1^ of the other EOs) [40,41] (Table 2). The EO from the inflorescences of the cultivar Felina 32 was also found to be highly toxic (LD_90_ of only 6.2 μL L^−1^) against the adults of the potato-peach aphid (*Myzus persicae*) [41] (Table 2). Additionally, an EO obtained from the inflorescences of three Polish hemp cultivars was shown to be effective against ornamental plant pests, such as the foxglove aphid (*Aulacorthum solani*) and the two-spotted spider mite (*Tetranychus urticae*), causing 100% and 98.72% mortality, respectively, at only 0.1% concentration [48] (Table 2).

The toxic activity of hemp EOs against insects and parasites observed in the reported studies may be attributed to α-pinene, myrcene, β-caryophyllene, α-humulene and terpinolene that, although at different percentages, have always been detected as the most abundant compounds in the EOs tested (Table 2). This assumption is supported by the numerous observations that, when used as pure compounds, these molecules showed high toxicity against insects and other pests. By way of example, α-pinene was found to be toxic against the mosquitos *A. aegypti* [49] and *Culex pipiens molestus* [50], and together with β-caryophyllene demonstrated excellent toxicity against *M. persicae* [51]. A toxic activity against *A aegypti* was also shown by myrcene [52], whereas β-caryophyllene and α-humulene were found to be highly toxic against the tick *H. dromedarii* and the mite *D. gallinae*, which may explain the acaricidal activity shown by the hemp EO on these two ectoparasites [45]. Insecticidal and larvicidal activities were also widely demonstrated for terpinolene [53].

Regarding the mechanism underlying the toxic activity of the EOs against insects and other parasites, several studies have demonstrated that EOs and pure terpenes act by targeting the insect nervous system, thus causing paralysis and death. The most widely studied mechanism is the inhibition of the acetylcholinesterase activity, which is crucial for neuronal transmission [54]. However, acetylcholinesterase inhibition does not seem the only neurotoxic action of EOs, and other mechanisms have been proposed through which the EO can modulate the neuronal activity of insects. Of these, the most relevant are the ability of EO and its components to act as positive allosteric modulators of GABA receptors and as agonists of the octopamine receptors, the latter being specific receptors for invertebrates including insects [54].

**Table 2:** Representative studies on the efficacy of hemp essential oils (EOs) as biopesticides.

Cultivar/Tissue	Composition	Target Organism	Effect	Ref.
*Insects harmful to humans*				
EO from hemp inflorescences	46.5–58.7% monoterpenes (2.2–4.0% α-pinene, 17.1–27.5% myrcene, and 14.0–17.0% limonene) and 35.6–48.6% sesquiterpenes (6.2–7.6% β-caryophyllene and 2.6–2.9% α-humulene)	Yellow fever mosquito (*Aedes aegypti*)	Biting deterrent activity comparable to *N*,*N*-diethyl-meta-toluamide LC_50_ = 21.8–27.5 ppm and LC_90_ = 38.7–38.9 ppm	[38]
EO from hemp inflorescences of cv. Futura 75	37.9% monoterpenes (7.8% α-pinene, 11.3% myrcene, and 7.6% terpinolene), 47.7% sesquiterpenes (21.4% β-caryophyllene and 7.1% α-humulene), and 11.4% cannabinoids (11.1% CBD)	Southern house mosquito (*Culex quinquefasciatus*) Housefly (*Musca domestica*)	LC_50_ = 124.5 μL L^−1^ and LC_90_ = 199.1 μL L^−1^ for *C. quinquefasciatus* larvae LD_50_ = 122.1 μg adult^−1^ and LD_90_ = 212.9 μg adult^−1^ for *M. domestica* adults	[40]
EO from hemp leaves of cv. Futura 75	5.3% monoterpenes (2.0% α-pinene and 0.9% myrcene), 75.0% sesquiterpenes (26.1% β-caryophyllene and 8.9% α-humulene), and 10.2% cannabinoids (10.0% CBD)		LC_50_ = 152.3 μL L^−1^ and LC_90_ = 410.3 μL L^−1^ for *C. quinquefasciatus* larvae LD_50_ = 305.2 μg adult^−1^ and LD_90_ = 428.7 μg adult^−1^ for *M. domestica* adults	
EO from hemp inflorescences of cv. Felina 32	54.2% monoterpenes (16.4% α-pinene, 14.2% myrcene, and 9.6% terpinolene), 45.6% sesquiterpenes (23.8% β-caryophyllene and 8.3% α-humulene), and 0.1% CBD	Southern house mosquito (*C. quinquefasciatus*) Housefly (*M. domestica*)	LC_50_ = 252.5 μL L^−1^ and LC_90_ = 700.9 μL L^−1^ for *C. quinquefasciatus* larvae LD_50_ = 43.3 μg adult^−1^ and LD_90_ = 213.5 μg adult^−1^ for *M. domestica* adults	[41]
EO from hemp inflorescences of cv. Kompolti in pure form and as nanoemulsion	60.0% monoterpenes (16.9% α-pinene, 6.7% β-pinene, 18.9% myrcene, 4.4% limonene, and 8.1% terpinolene), 37.7% sesquiterpenes (20.4% β-caryophyllene and 6.1% α-humulene), and 0.2% CBD	Southern house mosquito (*C. quinquefasciatus*)	LC_30_ = 35.5 ppm, LC_50_ = 56.8 ppm, LC_90_ = 142.3 ppm, and natality reduced by 40.4% at LC_30_ dose for the pure form LC_30_ = 46.8 ppm, LC_50_ = 72.2 ppm, LC_90_ = 207.2 ppm, and natality reduced by 45.1% at LC_30_ dose for the nanoemulsion	[42]
EOs from hemp inflorescences of cv. Felina 32	44.3% monoterpenes (15.1% α-pinene and 11.8% myrcene), 54.6% sesquiterpenes (34.8% β-caryophyllene and 11.4% α-humulene), and 0.1% cannabinoids	Asian malaria mosquito (*Anopheles stephensi*) African malaria mosquito (*Anopheles gambiae*)	82.7% and 100% mortality in larvae and pupae, respectively, of *A. stephensi* at 100 ppm 91.1% and 84.9% mortality in larvae and pupae, respectively, of *A. gambiae* at 100 ppm	[43]
EO from female hemp inflorescences of cv. Carmagnola Selezionata (CS)	68.3% monoterpenes (11.4% α-pinene, 24.3% myrcene, and 13.5% terpinolene), 29.1% sesquiterpenes (19.3% β-caryophyllene and 6.4% α-humulene), and 0.2% cannabinoids		90.2% and 94.2% mortality in larvae and pupae, respectively, of *A. stephensi* at 100 ppm 91.6% and 79.6% mortality in larvae and pupae, respectively, of *A. gambiae* at 100 ppm	
EO from male hemp inflorescences of cv. Carmagnola Selezionata (CS)	28.2% monoterpenes (8.0% α-pinene and 10.6% myrcene) and 71.8% sesquiterpenes (47.2% β-caryophyllene and 15.1% α-humulene)		89.8% and 90.5% mortality in larvae and pupae, respectively, of *A. stephensi* at 100 ppm 89.8% and 79.7% mortality in larvae and pupae, respectively, of *A. gambiae* at 100 ppm	
Hemp EO purchased from Assocanapa (Torino, Italy)	58.6% monoterpenes (7.7% α-pinene, 22.9% myrcene, and 12.0% terpinolene) and 39.0% sesquiterpenes (18.7% β-caryophyllene and 6.2% α-humulene)	Asian tiger mosquito (*Aedes albopictus*) Freshwater bladder snail (*Physella acuta*)	LC_50_ = 301.560 μL L^−1^, LC_90_ = 693.999 μL L^−1^, and 81.97% mortality at 500 μL L^−1^ for *A. albopictus* larvae LC_50_ = 35.370 μL L^−1^, LC_90_ = 46.691 μL L^−1^, and 100% mortality at 100 μL L^−1^ for *P. acuta* adults	[44]
*Insects harmful to animals*				
EO from hemp inflorescences of cv. Felina 32	54.2% monoterpenes (16.4% α-pinene, 14.2% myrcene, and 9.6% terpinolene), 45.6% sesquiterpenes (23.8% β-caryophyllene and 8.3% α-humulene), and 0.1% CBD	Poultry red mite (*Dermanyssus gallinae*) Camel tick (*Hyalomma dromedarii*)	LC_50_ = 47.1 μg mL^−1^ and LC_90_ = 493 μg mL^−1^ for *D. gallinae* adults LC_50_ = 73 μg mL^−1^, LC_90_ = 517 μg mL^−1^, and egg hatching rate reduced by 90% at 50 μg mL^−1^ for *H. dromedarii* larvae	[45]
Hemp EO obtained from Canapse	1.246% α-pinene, 5.5975% myrcene, 16.2067% γ-elemene, 7.0382% α-humulene, 5.2006% alloaromadendrene, 7.8595% selina-3,7(11)-diene, 10.0128% (E)-dauca-4(11),7-diene, and 14.1597% caryophyllene oxide	Cat flea (*Ctenocephalides felis felis*)	100% mortality at 200, 400, and 1600 µg cm^−2^ at egg, larval, and pupal stages, respectively 90% mortality at 2000 µg cm^−2^ at adult stage	[46]
EO from hemp leaves	23.45% myrcene, 16.24% limonene, and 16.64% β-caryophyllene	Varroa mite (*Varroa destructor*)	64.48%, 85.71%, and 95.4% acaricidal activity at 5%, 10%, and 15% EO, respectively	[47]
*Insects harmful to plants*				
EO from hemp leaves of cv. Futura 75	5.3% monoterpenes (2.0% α-pinene and 0.9% myrcene), 75.0% sesquiterpenes (26.1% β-caryophyllene and 8.9% α-humulene), and 10.2% cannabinoids (10.0% CBD)	Tobacco cutworm (*Spodoptera littoralis*)	LC_50_ = 112.8 μL L^−1^ and LC_90_ = 221.5 μL L^−1^	[40]
EO from hemp inflorescences of cv. Futura 75	37.9% monoterpenes (7.8% α-pinene and 11.3% myrcene), 47.7% sesquiterpenes (21.4% β-caryophyllene, 7.1% α-humulene, and 7.6% terpinolene), and 11.4% cannabinoids (11.1% CBD)		LC_50_ = 65.8 μL L^−1^ and LC_90_ = 89.3 μL L^−1^	
EO form hemp inflorescences of cv. Felina 32	54.2% monoterpenes (16.4% α-pinene, 14.2% myrcene, and 9.6% terpinolene), 45.6% sesquiterpenes (23.8% β-caryophyllene and 8.3% α-humulene), and 0.1% CBD	Tobacco cutworm (*Spodoptera littoralis*) Potato-peach aphid (*Myzus persicae*)	LC_50_ = 152.3 μL L^−1^ and LC_90_ = 313.1 μL L^−1^ for *S. littoralis* larvae LD_50_ = 3.5 μg adult^−1^ and LD_90_ = 6.2 μg adult^−1^ for *M. persicae*	[41]
EO form hemp inflorescences of cvs. Beniko, Bialobrzeskie and Silesia	9.76% α-pinene, 18.45% myrcene, 6.38% ocimene, 7.40 terpinolene, and 35.58% β-caryophyllene	Foxglove aphid (*Aulacorthum solani*) Two spotted spider mite (*Tetranychus urticae*)	23.87%, 57.33%, and 100.00% mortality at 0.02, 0.05 and 0.10% EO, respectively, for *A. solani* 71.14%, 79.80%, and 98.72% mortality at 0.02, 0.05, and 0.10% EO, respectively, for *T. urticae*	[48]

CBD, cannabidiol; EO, essential oil; LC, lethal concentration; LD, lethal dose.

### 3.3. Biopesticides Based on Hemp Extracts

Several studies have been carried out on the pesticidal properties of hemp extracts from leaves or inflorescences obtained by using different solvents [55]. As for the insects harmful to humans, the methanolic extract from leaves of cultivar Tango Kush showed a dose-dependent larvicidal effect against the yellow fever mosquito, reaching 100% mortality at 100 μL L^−1^ for both pyrethroid-susceptible and pyrethroid-resistant strains [56] (Table 3). When the extract was dried and the crude residue was partitioned between methanol and hexane, the larvicidal activity was detected only in the methanolic fraction. The analysis of the phytochemical profile revealed that CBD was the predominant component in the hemp extract and that 80% of this compound was recovered in the methanolic fraction. This prompted the authors to hypothesize that CBD was the main responsible for the observed larvicidal activity. Consistently, authentic CBD produced a concentration-dependent mortality in the mosquito larvae that was indistinguishable from the hemp leaf extract standardized for CBD concentration.

Extracts from hemp inflorescences and leaves were also tested against plant pathogens. The ethanolic extract from the inflorescences of the cultivar Cherry Dwarf, containing CBD as the main compound, was found to be toxic against the bacteria *Pseudomonas syringae* pv. *tomato*, *P. syringae* pv. *tabaci*, and *Erwinia carotovora*, which cause diseases in tomato, tobacco, and other crops, achieving 100% growth inhibition at concentrations ranging from 6.5 to 12.5 mg mL^−1^ [57] (Table 3). Also, an ethanolic extract obtained from the lateral inflorescences of the cultivar Futura 75, in which the polyphenolic component predominated, showed a dose-dependent antifungal activity against fungi responsible for various plant diseases, inhibiting growth up to 75.42–84.79% for *Alternaria alternata*, *Botrytis cinerea*, *Colletotrichum coccodes*, and *Trichoderma koningii*, and up to 38.80–72.95% for the *Fusarium* spp. [58] (Table 3).

The ethanolic extract from inflorescences of the cultivar Helena, characterized by the presence of phenolic compounds (4.71 mg g^−1^) mainly represented by flavonoids (4.40 mg g^−1^), was found to be effective against the Indian meal moth (*Plodia interpunctella*), a major pest of stored seeds and derived products [59] (Table 3). At the highest concentration tested (2%), the extract determined a significantly reduced female fecundity (−79%), a prolonged insect’s developmental duration (+37%), and up to −81% emerged moths (Table 3).

As emerged from the aforementioned studies, the phytochemical profile of the methanolic and ethanolic hemp extracts includes different classes of bioactive compounds, mainly cannabinoids, terpenes, phenols and flavonoids, which can all contribute to the toxic action of the extract. The potential mechanisms of action of cannabinoids and terpenes have already been described above. As for the phenolic compounds, evidence exists that, when used in their pure form, these compounds adversely affect the growth, feeding, oviposition and fecundity of a wide number of insects responsible for plant diseases [60], as well as insects harmful for animal and human health [61]. Similar to terpenes, these compounds act on the nervous system of the insect by negatively affecting the acetylcholinesterase activity [60,62]. In addition, evidence has been reported on the flavonoid ability to inhibit insect digestive enzymes, such as amylases, glycosidases, proteases, and lipases, which would be responsible for the antifeeding activity of this class of compounds, and to induce oxidative stress through the modulation of the detoxifying enzyme activities [60]. Phenolic compounds have also been reported to damage insect DNA by binding covalently to it and causing its fragmentation [63].

**Table 3:** Representative studies on the efficacy of hemp extracts as biopesticides.

Cultivar/Tissue	Composition	Target Organism	Effect	Ref.
*Insects harmful to humans*				
1.2–100 ppm methanolic extract from hemp leaves of cv. Tango Kush	CBD as the most abundant compound	Pyrethroid-susceptible (PS) and pyrethroid-resistant (PR) strains of yellow fever mosquito (*Aedes aegypti*)	LC_50_ = 4.3 and 4.4 μL L^−1^ for PR and PS strain, respectively	[56]
*Pathogens harmful to plants*				
0.19–100 mg mL^−1^ ethanolic extract from hemp inflorescences of cv. Cherry Dwarf	0.692% CBD, 0.196% THC, 0.186% nerolidol 2, 0.167% neryl acetate, 0.133% nerolidol 1, 0.105% α-bisabolene, 0.082% CBN, 0.081% β-bisabolene, 0.061% α-caryophyllene, 0.046% β-caryophyllene, and 0.045% limonene	*Pseudomonas syringae* pv. *tomato*, *P. syringae* pv. *tabaci*, *Erwinia carotovora*	81.6% and 100% growth inhibition at 3.13 and 6.5 mg L^−1^ extract, respectively, for *P. syringae* pv. *tomato* 97% and 100% growth inhibition at 3.13 and 12.5 mg L^−1^ extract, respectively, for *P. syringae* pv. *tabaci* 100% growth inhibition at 12.5 mg L^−1^ extract and less than 50% inhibition at lower concentrations for *E. carotovora*	[57]
5–20% ethanolic extract from lateral hemp inflorescences of cv. Futura 75	1.55 mg mL^−1^ flavonoids, 149.65 mg mL^−1^ polyphenols, 0.8 mg mL^−1^ CBD, and 0.4 mg mL^−1^ CBDA	*Alternaria alternata*, *Botrytis cinerea*, *Colletotrichum coccodes*, *Fusarium avenaceum*, *Fusarium culmorum*, *Fusarium graminearum*, *Fusarium oxysporum*, *Fusarium sporotrichioides*, *Trichoderma koningii*	Up to 75.42–84.79% growth inhibition for *A. alternata*, *B. cinerea*, *C. coccodes*, and *T. koningii* at 20% extract Up to 38.80–72.95% growth inhibition for *F. avenaceum*, *F. culmorum*, *F. graminearum*, *F. oxysporum*, and *F. sporotrichioides* at 20% extract Mycelium pigment disappearance and modification of the mycelium structure, altered intensity of fungal sporulation	[58]
*Insects harmful to stored products*				
0.5–2.0% ethanolic extract from hemp inflorescences of cv. Helena	2.29 mg g^−1^ total tannins, 4.44 mg g^−1^ total flavonoids, and 4.71 mg g^−1^ total phenolics (0.286 mg g^−1^ ferulic acid, 0.753 mg g^−1^ isovitexin, 0.287 mg g^−1^ rutin, 0.512 mg g^−1^ catechin, and 0.460 mg g^−1^ luteolin)	Indian meal moth (*Plodia interpunctella*)	Up to −79% female fecundity, +68% larval mortality, +37% mean developmental duration, and −81% emerged moths after feeding seeds treated with 2.0% extract	[59]

CBD, cannabidiol; CBDA, cannabidiolic acid; CBN, cannabinol; LC, lethal concentration; THC, Δ^9^-tetrahydrocannabinol.

## 4. Cosmetics

Evidence has been reported on the existence of the ECS in several types of cells and organs of the skin including epidermal keratinocytes, melanocytes, sebocytes, mast cells, fibroblasts, and hair follicles [8]. The skin produces different types of endocannabinoids, among which the most studied are 2-arachidonoylglycerol (2-AG) and N-arachidonoylethanolamine (AEA). The primary targets of these molecules in the skin are the two classic CB1 and CB2 receptors, but they also interact with Transient Receptor Potential Vanilloid (TRPV) receptors and Proliferator-Activated Receptors (PPAR) [8]. The primary physiological role of the cutaneous ECS appears to be the constitutive control of skin cell proliferation, differentiation, and survival, and the alteration of this delicate equilibrium may facilitate the development of disorders in the skin and hair growth [64]. Given the significant role that ECS plays in skin homeostasis, several studies have been carried out to assess whether the use of cannabinoid-based products can be effective in treating specific conditions or improving the overall skin and hair aesthetic and health. These studies have also included investigations on the safety of cannabinoid-based formulations in cosmetics, which have shown that topical application of non-psychoactive cannabinoids presents a low toxicological risk. At the low concentrations typically used in cosmetic products, these compounds have been shown to be safe for skin cells in vitro [65] and to not induce irritation, sensitization, or phototoxicity when applied to human skin [66,67]. Furthermore, percutaneous absorption studies indicate that topically applied cannabinoids undergo limited systemic absorption, insufficient to cause systemic toxicity [68].

### 4.1. Skin Aging

Skin aging is a degenerative process triggered by both internal (e.g., age, genetics, and hormones) and external (e.g., sun exposure, and tobacco smoking) factors, which lead to structural and physiological alterations in the skin resulting in undesirable appearance changes such as wrinkles, loss of elasticity, and xerosis [69].

Gerasymchuk and coworkers [70] used skin fibroblasts prematurely aged by exposure to hydrogen peroxide to assess the anti-aging effects of cannabinoids (Table 4). The authors observed that both CBD and THC at a concentration of 2 μM reduced senescent-associated morphological changes in skin cells, potentiated cellular viability and proliferation, upregulated the production of extracellular matrix (ECM) components, and downregulated the metalloproteinases responsible for their degradation [71]. In another study, the authors used the same model to assess the anti-aging effect of cannabinoids in combination with nutrient signaling modulators, such as metformin, resveratrol, and rapamycin [72] (Table 4), which are among the best-known regulators of processes involved in age-related diseases [73]. The results obtained revealed that CBD and THC potentiated the ability of triacetylresveratrol to ameliorate nuclear architecture and activate pathways associated with cell growth, metabolic activity, and anti-aging mechanisms; no effects were observed for the combination of cannabinoids with metformin, whereas rapamycin was found to inhibit cell viability [72].

CBD and two synthetic cannabinoids, S-88745 and S-91253, were tested by Chen and coworkers [74] for their anti-wrinkle properties in human fibroblasts damaged by UV radiation (Table 4). CBD, and even more so its derivatives, at concentrations between 0.16 μM and 4 μM were found to inhibit apoptosis, counteract the increase in ROS production and the decrease in collagen, elastin, and fibronectin induced by UV radiation. This was probably ascribable to the anti-inflammatory properties of these compounds. Indeed, the same authors demonstrated that CBD and its derivatives were able to downregulate the expression of pro-inflammatory interleukin-6, cyclooxygenase-2, and nitric oxide synthase in a macrophage cell line [74].

The anti-inflammatory properties of CBD were demonstrated also by Cohen and coworkers [75] on both human keratinocytes and skin tissue culture aged by UVB exposure (Table 4). The authors demonstrated that 10 mg L^−1^ CBD were able to inhibit the secretion of prostaglandin E2 (PGE2) and interleukin-8 (IL-8), two primary inflammatory agents associated with photoaging, in human keratinocytes; the inhibitory property of CBD was potentiated when it was applied in combination with 10 mg L^−1^ eicosapentaenoic acid (EPA), which is known to have beneficial cosmetic and therapeutic properties [76]. Consistently, a reduction in the secretion of PGE2 and IL-8 was also observed in the UVB-damaged human skin tissue after application of a topical cream formulation based on both active ingredients at a concentration of 0.1%. Histological examination also revealed that cream application resulted in an increased ECM, which restored the normal skin architecture. The authors also used the topical cream formulation to carry out a clinical trial and observed a time-dependent reduction of wrinkles and an improvement in skin firmness and elasticity. Based on questionnaire responses, almost all patients expressed high levels of satisfaction with the product’s outcomes [75] (Table 4).

Consistent with what was observed with purified cannabinoids, in vitro experiments demonstrated that extracts form hemp herbs (leaves, inflorescences and stem), which contained CBD as major component together with polyphenols and flavonoids, inhibited metalloproteinases such as collagenase and elastase [77], while an extract from hemp seed paste by-product exhibited its inhibitory activity against collagenase but not elastase [78].

### 4.2. Skin Moisture

Evidence exists on the ability of CBD to prevent water loss from the skin and preserve skin hydration. In this regard, different mechanisms have been proposed. Ikarashi and coworkers [79] applied a 1% CBD solution to the skin of hairless mice and observed an increase in the dermal water content (Table 4). The authors also observed an increase in the aquaporin-3 (AQP3) at both mRNA and protein level. AQP3 is an aquaporin highly expressed in the basal layer of keratinocytes in the mammalian epidermis where it is responsible for the transport of glycerol and water [80]. Studies carried out on mouse models revealed that the protein level of AQP3 was significantly lower in aged compared to young mice, which suggested that this protein was the cause of age-related skin dryness [81]. Based on these previous observations, it is feasible that the CBD ability to preserve skin hydration may be related to its ability to up-regulate the AQP3 protein expression.

A different mechanism was proposed by Łuczaj and coworkers [82] (Table 4). The authors observed that the topical application of 2.5% CBD to the skin of nude rats irradiated with UVA or UVB induced a decrease in the phospholipase A_2_ (PLA_2_) activity and, consequently, in the lysophospholipid content. It is known that the PLA_2_ activity increases as a consequence of the increase in oxidized phospholipids, which are neutralized by the enzyme through the cleavage of the oxidized residue and the release of the lysophospholipid [83]. This suggests the ability of CBD to counteract the oxidative stress induced by UV radiation and prevent the oxidative modification of phospholipids. Additionally, CBD induced an increase in sphingomyelinase activity with a consequent activation of the sphingomyelin catabolism, which determined an increase in the levels of ceramides [82] (Table 4). Together with cholesterol and free fatty acids, ceramides are the major components of the inter-corneocyte lipids and play a major role in the skin barrier function preventing water evaporation [84]. Consistently, a decrease in ceramide levels was observed in the stratum corneum of patients with various skin diseases, and this decrease was considered to be responsible for the dry skin that these conditions are known to cause [85].

A moisturizing effect on skin was also observed in a clinical study that used hemp extracts containing CBD as the major component (130–150 mg g^−1^), followed by phenolic compounds (42.5–52.3 mg g^−1^) and flavonoids (8.1–10.4 mg g^−1^) [77] (Table 4). A hydrogel containing 0.5% or 1.0% hemp extracts was applied to the skin after the cleaning process with 1% sodium lauryl sulfate (SLS), one of the most often used cleaning ingredients in cleansing cosmetic formulations. SLS significantly decreased skin moisture by 11–15% compared to untreated skin, but subsequent application of the hydrogel containing 0.5% extract restored normal skin conditions; an even stronger effect was obtained with 1.0% extract that increased the skin moisture by up to 10% compared to normal skin.

### 4.3. Skin Hyperpigmentation

Cutaneous hyperpigmentation is characterized by spots that become darker than the surrounding skin. It is due to an abnormal overproduction of melanin, the pigment that gives skin its color. One of the most common approaches for control of skin hyperpigmentation involves the inhibition of tyrosinase, the enzyme that catalyzes the key step of melanogenesis and whose activity regulates the amount of melanin accumulated in the skin [86]. Therefore, the cosmetic industry is increasingly interested in natural ingredients and compounds that inhibit tyrosinase activity.

Chen and coworkers [74] tested the ability of CBD and the two synthetic cannabinoids, S-88745 and S-91253, to control the melanogenesis induced by the addition of α-melanocyte stimulating hormone to murine melanoma cells (Table 4). The authors observed that both CBD and the synthetic compounds at concentrations ranging between 0.0256 μM and 0.64 μM were able to reduce the intracellular melanin content (Table 4). By using the same murine model, Gaweł-Beben and coworkers [87] demonstrated that not only CBD but also minor cannabinoids, namely, CBG and CBN at 5 μg mL^−1^ concentration reduced the intracellular melanin content (up to 67.87%) and its release (up to 45.14%), whereas no effect was observed for CBC. Consistently, in both these studies, as well as in other studies [67,88], it was demonstrated that both cannabinoids and their synthetic derivatives were able to inhibit the activity of mushroom and murine tyrosinase in vitro (Table 4). Notably, potent tyrosinase inhibitors were obtained by Peretz and coworkers [88], who synthesized CBD-based thiosemicarbazone analogs, and observed that while CBD exerted a minimal inhibitory activity against mushroom tyrosinase with an IC_50_ higher than 100 μM, all the five derivatives tested strongly inhibited the enzyme with an IC_50_ that ranged between 22.41 μM and 42.16 μM (Table 4), which was comparable or even lower than that detected for kojic acid (IC_50_ = 35.33 μM), a potent and extensively studied tyrosinase inhibitor [89].

### 4.4. Hair Growth

The effect of cannabinoids on hair growth is complex and depends on their concentration and the receptor to which they bind. Evidence exists that the activation of CB1, TRPV1, and TRPV4 receptors negatively affects hair growth by reducing keratinocyte proliferation, inducing premature regression of the hair follicles, and preventing the hair shaft elongation [90,91]. In this regard, a study carried out by Telek and coworkers [92] on human hair follicles demonstrated that THC, acting as agonist of the CB1 receptor, was able to induce the inhibition of the hair follicle growth and the shaft elongation in a dose-dependent manner (Table 4). On the other hand, Szabò and coworkers [93] observed that CBD used at submicromolar concentration (0.1 μM) stimulated hair shaft elongation in human hair follicles, whereas micromolar concentration (10 μM) inhibited hair shaft production (Table 4). Investigations carried out by the same authors on outer root sheath keratinocytes revealed that the inhibition of the hair growth observed at micromolar CBD concentration was due to the activation of the TRPV4 receptor, which is known to promote catagen phase in human hair follicles [94], whereas the stimulation of the hair shaft elongation observed at submicromolar CBD concentration was due to the ability of CBD to reduce the intrafollicular production of the proinflammatory cytokines in an adenosine receptor-dependent manner. However, the CBD modulation of other receptors cannot be excluded. Indeed, CBD could also stimulate hair growth through the negative allosteric modulation of the CB1 receptor, and/or its interaction with the TRPV1 receptor. In this regard, it is known that, although CBD acts as an activator of the TRPV1 receptor, this may become rapidly desensitized upon activation by CBD, and this could lead hair growth stimulation [95].

Overall, these observations suggest the potentiality of CBD in the treatment of both hair loss and unwanted hair growth. In this regard, a case series study carried out on 35 subjects with androgenetic alopecia demonstrated that an oil formulation obtained from hemp inflorescences applied at a dose of about 3–4 mg day^−1^ CBD for 6 months increased the hair number in the temporal area by 74.1% in men and 55.2% in women, and in the vertex area by 120.1% in men and 64.9% in women [96] (Table 4). Better results were obtained with a hemp oil formulation obtained from a whole plant extract containing CBD as a major component and other minor cannabinoids, such as CBDV and THCV [97] (Table 4). Its application at a dose of 33 mg day^−1^ for 6 months determined an average increase in the hair number of 246% in men and 127% in women. Although the mechanism of action of CBDV and THCV are not known, the authors hypothesized that these cannabinoids most likely functioned as CB1 receptor antagonists and TRPV1 receptor agonists.

**Table 4:** Representative studies on the efficacy of cannabinoids as skin and hair care agents.

Treatment	Target Tissue	Effect	Ref.
*Skin aging*			
2 μM THC or CBD in DMSO	Human neonatal foreskin fibroblasts (CCD-1064Sk) treated for 1 h with 25 μM concentration of hydrogen peroxide	Maintenance of normal cell and nucleus morphology Increase in cell viability through the regulation of proteins involved in cell cycle and senescence Maintenance of the ECM through the increase in the collagen, elastin, hyaluronan synthase levels and decrease in the metalloproteinase levels	[70]
2 μM THC or 2 μM CBD in combination with 500 μM metformin, 10 μM triacetylresveratrol (TRSV), or 5 μM rapamycin in DMSO	Human neonatal foreskin fibroblasts (CCD-1064Sk) treated for 1 h with 25 μM concentration of hydrogen peroxide	Amelioration of nuclear architecture, downregulation of cell growth inhibitors, upregulation of pro-longevity sirtuins, CB1 and CB2 receptors, and collagen and elastin in CBD+TRSV and THC+TRSV treatments	[72]
0.16, 0.8 and 4 μM CBD or CBD derivatives S-88745 and S-91253 in aqueous solution	Human foreskin fibroblasts (HFF-1) damaged by UV radiation	61.2%, 89.8%, and 93.6% ROS decrease at 0.8 μM CBD, S-88745 and S-91253, respectively 53%, 64%, and 61.4% apoptosis inhibition at 0.16 μM CBD, and 4 μM S-88745 and S-91253, respectively 27.7%, 19.0%, and 40.8% collagen increase at 4 μM CBD, and 0.16 μM S-88745 and S-91253, respectively 38.8%, 17.9%, and 63% elastin increase at 0.16 μM CBD, S-88745 and S-91253, respectively 26.7% fibronectin increase at 0.16 μM S-91253	[74]
10 mg L^−1^ CBD or 10 mg L^−1^ CBD + 10 mg L^−1^ eicosapentaenoic acid (EPA) for keratinocyte cells Topical cream containing 0.1% CBD + 0.1% EPA for skin tissue and clinical study	Human keratinocyte cells (HaCaT) damaged by UVB exposure Human skin tissue damaged by UVB exposure Clinical study on thirty-four female subjects aged between 45 and 65 years	Decrease in the production of prostaglandin E2 and interleukin-8 both in keratinocyte cells and skin tissue Increase in cell viability and restoration of normal ECM architecture in skin tissue Disappearance of wrinkles, and improvement in skin elasticity, skin hydration, firmness, and clear reduction in aging signs in the female subjects	[75]
*Skin moisture*			
1% CBD in aqueous solution	Seven-week-old male HR-1 hairless mice	Increase in dermal water content Increase in gene expression and protein level of aquaporin-3 (AQP3)	[79]
2.5% CBD *w*/*w* in petrolatum	8–9-week-old male nude rats (RH-FOXN1RNU) irradiated with UVA or UVB	Decrease in phospholipase A_2_ activity and lysophospholipid content Increase in phosphatidylserine and phosphatidylethanolamine content Increase in sphingomyelinase activity and increase in ceramide content	[82]
Hydrogel containing 0.5% or 1%: magnetically stirred hemp extract (MAE) (130.0, 42.5, and 8.1 mg g^−1^ CBD, TPC, and TFC, respectively) or ultrasound assisted hemp extract (UAE) (150.0, 51.3, and 10.4 mg g^−1^ CBD, TPC, and TFC, respectively)	Forearm skin of fifteen 28–36-year-old volunteers washed with 1% sodium lauryl sulfate (SLS)	Restoration of the skin moisture measured before washing with 1% SLS after treatment with 0.5% MAE or UAE hydrogel 5% and 10% increase in skin moisture compared to that measured before washing with 1% SLS after treatment with 1.0% MAE and 1.0% UAE hydrogel, respectively Rebuilding of the skin hydrolipid barrier damaged by washing with 1% SLS	[77]
*Skin hyperpigmentation*			
0.0256, 0.128, and 0.64 μM CBD, or CBD derivatives S-88745 and S-91253 in aqueous solution	Murine melanoma cells (B16F10) treated with α-melanocyte stimulating hormone (α-MSH)	Decrease in melanin content Inhibition of tyrosinase activity	[74]
2.5 and 5.0 μg mL^−1^ CBD, CBG, CBN, or CBC in DMSO	Murine melanoma cells (B16F10) treated with α-MSH	45.14%, 29.76%, and 34.14% decrease in melanin release at 5.0 μg mL^−1^ CBD, CBG, and CBN, respectively 67.87%, 61.25%, and 60.59% decrease in melanin content at 5.0 μg mL^−1^ CBD, CBG, and CBN, respectively	[87]
50, 100, and 200 μg mL^−1^ CBD, CBG, CBN, or CBC in DMSO	Commercial mushroom tyrosinase and tyrosinase from murine melanoma cells	Inhibition of mushroom tyrosinase activity at 100 and 200 μg mL^−1^ CBG, CBN, and CBC Inhibition of murine tyrosinase activity at 50 and 200 μg mL^−1^ CBN	
5, 10, 50, and 100 μM CBD or CBG in 60% ethanol	Commercial tyrosinase	Up to 38.36% and 86.84% decrease in mushroom tyrosinase activity for CBD and CBG, respectively	[67]
10–150 μM CBD-based thiosemicarbazone analogs in DMSO	Commercial mushroom tyrosinase	IC_50_ > 100 μM for CBD IC_50_ ranging between 22.41 and 42.16 μM for the thiosemicarbazone analogs	[88]
*Hair growth*			
2 and 20 μM THC	Human hair follicles from women undergoing face-lift surgery	Dose-dependent inhibition of hair shaft elongation Suppression of hair follicle keratinocyte proliferation Increase in keratinocyte apoptosis and premature catagen development	[92]
0.1 and 10 μM CBD	Human hair follicles Outer root sheath keratinocytes	Increase in hair shaft elongation in hair follicles treated with 0.1 μM CBD Decrease in hair shaft elongation in hair follicles treated with 10 μM CBD Downregulation of cytokine genes in keratinocytes treated with 0.1 μM CBD Activation of the TRPV4 receptor in keratinocytes treated 10 μM CBD	[93]
Hemp oil formulation containing inflorescence extract (10.78% CBD and 0.21% THC) applied at a dose of 3–4 mg day^−1^ CBD	Twenty-eight males and seven females with androgenetic alopecia	74.1% and 55.2% hair count increase in the temporal area of men and women, respectively, treated with 3–4 mg day^−1^ CBD for six months 120.1% and 64.9% hair count increase in the vertex area of men and women, respectively, treated with 3–4 mg day^−1^ CBD for six months	[96]
Hemp oil formulation containing whole plant extract (60% CBD, 12.63% CBDV, 3.71% THCV, 0.86% CBG, and 0.18% THC) applied at a dose of 33 mg day^−1^ hemp extract	Fifteen males and sixteen females with androgenetic alopecia	246% and 127% hair count increase in men and women, respectively, treated with 33 mg day^−1^ extract for six months	[97]

CBC, cannabichromene; CBD, cannabidiol; CBDV, cannabidivarin; CBG, cannabigerol; CBN, cannabinol; DMSO, dimethyl sulfoxide; ECM, extracellular matrix; ROS, reactive oxygen species; TFC, total flavonoid content; THC, Δ^9^-tetrahydrocannabinol; THCV, tetrahydrocannabivarin; TPC, total phenolic content.

## 5. Foods and Dietary Supplements

*C. sativa* has been a valuable dietary source for humans for thousands of years. The first evidence of its use was provided by the discovery of hemp seeds in Chinese tombs dating back to the third millennium before Christ [98]. Recently, the rediscovery of the health benefits of hemp has led to numerous studies aimed at evaluating the use of hemp seeds, leaves and inflorescences as ingredients in foods and dietary supplements.

Currently, in many countries, hemp seeds are the only part of the hemp plant that is explicitly allowed for the production of foods and dietary supplements because they do not naturally contain cannabinoids [99,100,101,102,103,104,105] (Table 5). In particular, in the United States (US), hulled hemp seeds, along with hemp seed protein powder and hemp seed oil, are generally recognized as safe (GRAS) by the Food and Drug Administration (FDA) for use in human foods [99]. In the European Union (EU), hemp seeds and their derived products are recognized as a traditional food and are not subject to the novel food regulations, but their total THC (the sum of THC and THCA) levels due to contamination during harvesting and processing are tightly regulated and cannot exceed 3 mg kg^−1^ in hemp seeds and flour and 7.5 mg kg^−1^ in hemp seed oil [100].

By contrast, the use of leaves and inflorescences as food ingredients is prohibited in most countries [101,102,103,104,105,106,107] (Table 5). In 2018, the Food and Drug Administration (FDA) has approved the anticonvulsant drug Epidiolex, which contains a highly purified form of CBD extracted directly from hemp plant; as an active ingredient in a drug, purified CBD and ingredients derived from parts of the hemp plant that contain CBD cannot be legally used as additives in foods or supplements in the US [106]. On the other hand, the European Commission (EC) has classified hemp extracts and derived products containing cannabinoids as novel foods [107]. This implies that CBD-containing foods and supplements must pass a rigorous safety evaluation by the European Food Safety Authority (EFSA) before receiving EC approval as novel foods and being legally marketed in the EU. However, EFSA’s current stance is that the safety of CBD as a novel food cannot be established due to gaps in the available data on the potential hazards associated with CBD consumption. So, to date, none of the CBD-based products submitted for the recognition as a novel food have yet been authorized in the EU.

**Table 5:** Global regulatory overview of hemp seed and CBD-infused foods.

Region	Hemp Seed Foods	THC Limit in Hemp Seed Foods	CBD-Infused Foods	THC Limit in CBD-Infused Foods	Ref.
US	Allowed (GRAS)	No federal limit	Not federally allowed	—	[99,106]
EU	Allowed	≤3 mg kg^−1^ for hemp seeds and flour and ≤7.5 mg kg^−1^ for hemp seed oil	Classified as novel foods (no products approved yet)	No explicit legal limit	[100,107]
UK	Allowed	No explicit legal limit	Classified as novel foods	70 μg day^−1^	[101]
Canada	Allowed	≤10 μg g^−1^	Not allowed	—	[102]
Australia	Allowed	≤5 mg kg^−1^ for hemp seeds and flour, ≤0.2 mg kg^−1^ for hemp seed beverages, and ≤10 mg kg^−1^ for hemp seed oil	Not allowed	—	[103]
Japan	Allowed	≤10 ppm for hemp seed oil, ≤1 ppm for edibles and powder, and ≤0.1 ppm for aqueous solutions	Not allowed	—	[104]
China	Limited (no legal authorization)	No explicit law limit	Not allowed	—	[105]

### 5.1. Hemp Seeds

Hemp seeds are an excellent source of oil (25–35%), proteins (20–25%), and fiber (10–15%) [108]. The principal value of hemp seeds is in their fatty acid composition that, in comparison to other oil seeds, has the highest proportion of polyunsaturated fatty acids (PUFA) belonging to ω-3 and ω-6 classes. In addition, their proteins are well known for their digestibility and essential amino acid composition, whereas carbohydrates are largely composed of dietary fiber, mostly the insoluble type [109]. Hemp seeds are also a great source of many different phytochemicals, with tocopherols and phenolic compounds being the most abundant [110]. These two classes of compounds are responsible for the high antioxidant activity found in hemp seeds [111], which make them particularly suitable for food and nutraceutical applications. Hemp seeds are used as whole, for the extraction of oil or the production of flour, the latter used for the preparation of processed products such as pasta and bakery products.

#### 5.1.1. Oil

For its high content of essential fatty acids and phytochemicals, and its subtle and nutty flavor, hemp seed oil is a healthy alternative to other cooking oils, and it is also gaining popularity as a dietary supplement. As shown in Table 6, the lipid profile of hemp seed oil is represented by more than 70% PUFA, mainly represented by linoleic acid (38–58%) followed by α-linolenic acid (11–19%) [112,113,114,115,116,117]. Hemp seed oil also boasts a favorable ω-6:ω-3 ratio ranging between 2.6 and 3.9, which is ideal for human diet since it is known to prevent chronic conditions, e.g., cardiovascular disease, diabetes, obesity, cancer, and autoimmune diseases [118].

The unsaponifiable fraction of hemp seed oil also contains a variety of bioactive compounds, which include phytosterols and tocopherols. The total phytosterol content ranges approximately between 2200 and 5900 mg kg^−1^ oil [112,119], with β-sitosterol and campesterol being the most abundant, followed by stigmasterol [112,113,114,119] (Table 6). These naturally occurring plant compounds, which are structurally related to cholesterol, are of great interest since they are known to have cholesterol-lowering, anti-inflammatory, and antioxidant effects, in addition to immune system-boosting benefits [120]. The total amount of tocopherols detected in hemp seed oil is in the range of 114 and 1118 mg kg^−1^ oil [112,116,119,121] (Table 6), which is higher than most of the extra-virgin olive oils [122]. However, while α-tocopherol is the main isomer present in olive oil, γ-tocopherol is the most abundant in hemp seed oil representing more than 80% of total tocopherols (Table 6). Recent studies have demonstrated that γ-tocopherol has unique antioxidant, anti-inflammatory, and potential anti-cancer properties, thus making it a valuable component of a healthy diet [123]. Thanks to their antioxidant activity, phytosterols and tocopherols also play an important role in preserving the stability of hemp seed oil that, due to the high amount of PUFA, is very susceptible to oxidative deterioration during extraction, storage, and food preparation [124]. Oxidative stability is also favored by the presence of phenolic compounds and carotenoids, which although in lower amounts have still been detected in hemp seed oil. The total phenolic content (TPC) in hemp seed oil, evaluated as gallic acid equivalents (GAE), ranged from 2.1 to 267 mg 100 g^−1^ oil, with flavonoids, e.g., naringenin, catechin, epicatechin kaempferol and quercetin, being the predominant compounds [113,114,115,121] (Table 6). Total carotenoids were detected at levels ranging from 1.78 to 61 mg kg^−1^ [114,117,119] (Table 6). Due to their ability to protect chlorophylls from photo-oxidation, their presence is particularly important to prevent any color changes due to chlorophyll degradation. In addition, consumption of both phenolic compounds and carotenoids has beneficial effects on human health since, thanks to their antioxidant activity, these compounds are able to prevent the onset of various chronic diseases [125].

**Table 6:** Representative studies on the chemical composition of hemp seed oils.

Hemp Seed Oil	Phytochemicals	Other Compounds	Ref.
Hemp seed oil provided by Botanica Nutrients (Seville, Spain)	1905.07 mg kg^−1^ β-sitosterol, 505.69 mg kg^−1^ campesterol, 142.80 mg kg^−1^ Δ_5_-avenasterol, 100.23 mg kg^−1^ stigmasterol, and 2793.73 mg kg^−1^ total sterols 3.22 mg 100 g^−1^ α-tocopherol, 0.81 mg 100 g^−1^ β-tocopherol, 73.38 mg 100 g^−1^ γ-tocopherol, 2.87 mg 100 g^−1^ δ-tocopherol, and 80.28 mg 100 g^−1^ total tocopherols	55.05% linoleic acid, 16.70% α-linolenic acid, 3.40% γ-linolenic acid, and 75.46% total PUFA 3.5 ω-6:ω-3 ratio 167.59 mg kg^−1^ phytol, 80.52 mg kg^−1^ squalene, and 43.35 mg kg^−1^ waxes	[112]
Hemp seed oil extracted from seeds of cv. Fedora	530.4 mg kg^−1^ β-sitosterol, 117.4 mg kg^−1^ campesterol, 72.6 mg kg^−1^ Δ_5_-avenasterol, and 28.2 mg kg^−1^ stigmasterol 21 mg GAE kg^−1^ TPC	56.08% linoleic acid, 14.89% α-linolenic acid, 3.03% γ-linolenic, and 75. 03% total PUFA 3.9 ω-6:ω-3 ratio	[113]
Hemp seed oils extracted from seeds of cv. USO-31	80.76–90.75% β-sitosterol, 6.20–14.19% campesterol, and 2.88–5.05% stigmasterol 39.19–49.31 mg kg^−1^ α-tocopherol and 770.08–967.05 mg kg^−1^ γ-tocopherol 9.60–61.00 mg kg^−1^ total carotenoids 33.59–51.42 mg GAE kg^−1^ TPC 41.15–71.51 mg kg^−1^ CBD and 68.66–113.92 mg kg^−1^ CBN	56.85–58.04% linoleic acid, 15.68–15.86% α-linolenic acid, 2.99–4.09% γ-linolenic acid, and 75.66–78.34% total PUFA 3.81–3.91 ω-6:ω-3 ratio 12.80–125.51 mg kg^−1^ chlorophyll a and 14.55–23.29 mg kg^−1^ chlorophyll b	[114]
Hemp seed oil provided by Oil Seed Extractions Limited (Ashburton, New Zealand)	2.78 mg 100 g^−1^ α-tocopherol and 56.41 mg 100 g^−1^ γ-tocopherol 188.23 mg GAE 100 g^−1^ TPC and 19.50 mg QRC 100 g^−1^ TFC	56.85% linoleic acid, 18.76% α-linolenic acid, and 4.76% γ-linolenic acid 3.29 ω-6:ω-3 ratio 75.21 mg kg^−1^ total chlorophyll content	[115]
Hemp seed oils extracted from seeds purchased from a local market (Karai, Iran)	7.90–43.22 mg kg^−1^ α-tocopherol, 792.86–892.60 mg kg^−1^ γ-tocopherol, 31.85–35.48 mg kg^−1^ δ-tocopherol, and 832.61–971.30 mg kg^−1^ total tocopherols	55.07–55.30% linoleic acid, 18.09–18.50% α-linolenic acid, 0.60–1.01% γ-linolenic acid, and 74.2–74.4% total PUFA 2.97–3.05 ω-6:ω-3 ratio	[116]
Thirteen commercial hemp seed oils	14.6–53.0 mg kg^−1^ α-tocopherol, 594–967 mg kg^−1^ γ-tocopherol, and 19.6–50.3 mg kg^−1^ δ-tocopherol 2.53–33.93 mg kg^−1^ total carotenoids 4.25–91.60 mg kg^−1^ CBDA, 0.0–22.2 mg kg^−1^ CBD, 0.0–5.0 mg kg^−1^ THCA, and 0.0–5.29 mg kg^−1^ THC Terpenes including α-pinene, β-pinene, myrcene, limonene, and (*Z*)-β-ocimene	38.48–52.16% linoleic acid, 11.02–17.40% α-linolenic acid, 0.98–4.43% γ-linolenic acid, and 52.59–70.38% total PUFA 2.60–3.67 ω-6:ω-3 ratio	[117]
Four commercial hemp seed oils from Italy and four from Extra-European countries	345.14–813.8 mg kg^−1^ campesterol, 1510–4010 mg kg^−1^ β-sitosterol, 50.10–247.5 mg kg^−1^ stigmasterol, and 2199–5891 mg kg^−1^ total sterols 0.35–7.76 mg 100 g^−1^ α-tocopherol, 0.37–0.58 mg 100 g^−1^ β-tocopherol, 62.53–101.32 mg 100 g^−1^ γ-tocopherol, 1.40–3.51 mg 100 g^−1^ δ-tocopherol, and 65.50–111.80 mg 100 g^−1^ total tocopherols 1.78–2.61 μg g^−1^ carotenoids	24.9–52.2 μg g^−1^ chlorophyll a and 9.9–24.2 μg g^−1^ chlorophyll b 21.92–122.20 mg kg^−1^ total alcohols	[119]
Hemp seed oil extracted from seeds of cv. Finola	19.74 mg kg^−1^ α-tocopherol, 0.64 mg kg^−1^ β-tocopherol, 91.57 mg kg^−1^ γ-tocopherol, 2.09 mg kg^−1^ δ-tocopherol, and 114.04 mg kg^−1^ total tocopherols 267.5 mg GAE 100 g^−1^ TPC, 2780.4 mg QRC 100 g^−1^ TFC, 29.744 μg 100 g^−1^ naringenin, 10.157 μg 100 g^−1^ epicatechin, 5.284 μg 100 g^−1^ catechin, 6.501 μg 100 g^−1^ kaempferol-3-O-glucoside, 3.908 μg 100 g^−1^ quercetin-3-O-rutinoside, and 3.434 μg 100 g^−1^ kaempferol-3-O-rutinoside		[121]

CBD, cannabidiol; CBDA, cannabidiolic acid; CBN, cannabinol; GAE, gallic acid equivalents; PUFA, polyunsaturated fatty acids; QRC, quercetin equivalents; TFC, total flavonoid content; THC, Δ^9^-tetrahydrocannabinol; THCA, Δ^9^-tetrahydrocannabinolic acid; TPC, total phenolic content.

Finally, trace amounts of some monoterpenes (e.g., α-pinene, β-pinene, myrcene, limonene, and (*Z*)-β-ocimene) and cannabinoids (e.g., CBD, CBDA, THC, THCA, and CBN) were also detected in hemp seed oil [114,117] (Table 6). As mentioned above, these compounds are not produced in the seeds and are transferred into the oil during harvesting and processing following contact of the seeds with the inflorescences and leaves [12]. In this regard, Lindekamp and coworkers [126] observed that the contamination by cannabinoids varied among different oil samples and that the lowest levels were detected in oils obtained from dehulled seeds, thus underlining the importance of cleaning and peeling the seeds before oil pressing to avoid contamination.

#### 5.1.2. Flour

Due to the increasing consumer demand for food products enriched with valuable nutrients, the fortification of cereal-based foods with hemp seed flour has been growing in popularity recently. Hemp flour can be obtained by milling the whole hemp seeds, by grinding the hemp seed cake, the by-product obtained through the mechanical oil extraction, or by grinding the hemp seed meal, the by-product obtained after solvent oil extraction of the pressed cake. Hemp flour is gluten-free and provides higher proteins, fats, and fiber compared to wheat flour [127,128], thus making it a healthier option. The use of hemp flour as ingredient in cereal-based foods is related not only to its macronutrient content and composition, but also to its high content of bioactive compounds, mainly phenolic compounds, which are present at higher levels compared to wheat flour [127,128]. Consistently, pasta obtained by replacing a part of wheat flour with hemp seed cake flour had not only a higher content of proteins, lipids and minerals compared to traditional pasta, but also a higher content of phenolic compounds, which increased as the amount of hemp seed flour added to the dough increased, reaching levels from 20% to 340% higher than those observed in whole wheat flour pasta [127,129] (Table 7). Similar findings were reported for other cereal-based products. By way of example, 119–162% increase in TPC was detected in wheat bread supplemented with 15–50% partially defatted hemp seed flour [130], while bread supplementation with 5–40% flour from hemp seed cake led to 23–220% TPC increase [128,131] (Table 7). Also, replacement of corn flour with 20–60% hemp seed flour led to 41–143% higher TPC in biscuits [132] (Table 7). Consistent with the increase in TPC, all supplemented products showed a concomitant increase in the total antioxidant activity (TAA) compared to the corresponding traditional product (Table 7).

However, it should be noted that the addition of hemp seed flour also changed the physicochemical properties of the end-products (e.g., increase in pasta cooking time, decrease in bread and biscuits volume, and browning), as well as their sensory properties, which influenced the overall acceptability by consumers. From the data reported in Table 7, a general worsening of the physicochemical and organoleptic properties of the supplemented foods was observed as the amount of hemp seed flour added increased, although with differences among the various types of products. For this reason, it is essential to determine the optimal amount of hemp seed flour to add to each product in order to create a fortified food that also meets customer sensory preferences.

### 5.2. Inflorescences and Leaves

Despite existing regulatory constraints, an increasing number of dietary supplements and food products formulated with hemp leaves, inflorescences, or CBD extracts incorporated into various matrices have been introduced to the market in recent years.

#### 5.2.1. Hemp-Infused Foods

Beer is the most consumed alcoholic beverage in the world and is traditionally flavored with hop (*Humulus lupulus* L.), a close relative of *C. sativa*. This has prompted researchers to formulate new beers enriched with hemp inflorescences to enhance aroma. A mix of fresh inflorescences from cultivars Futura 75 and Uso 31 were used as flavoring agent in the preparation of an artisanal beer [133] (Table 8). The addition of the inflorescences at different stages of the brewing process determined a slight decrease in the non-terpene volatiles, in particular ethyl hexanoate (from 11.7% to 10.2%) and ethyl octanoate (from 32.2% to 24.7%), which represented the predominant volatiles in this product, and a concomitant increase in the monoterpenes (from 6.1% to 10.3%), which are abundant in the hemp inflorescences, with the highest increase observed for myrcene, whose concentration increased from 5.6% to 9.8%. A similar result was reported by Cárdenas-Pinto and coworkers [134], who analyzed three beers at different ethanol concentrations (3%, 6%, and 9%) added with hemp inflorescences after fermentation (dry-hemping) (Table 8). The authors reported an increase in both monoterpenes and sesquiterpenes in hemp beers compared to traditional beer (3% ethanol) and observed that the increase in monoterpenes was greater the higher the alcohol content (0.42 mg L^−1^ in control beer vs. 5.36, 6.12, and 6.62 mg L^−1^ in 3%, 6%, and 9% hemp beer, respectively), thus indicating that ethanol concentration in the beer positively influenced the extractability of these compounds from the hemp inflorescences. By contrast, CBDA decreased with increasing ethanol concentration moving from 44.5 ng mL^−1^ at 3% ethanol to 11.45 ng mL^−1^ at 9% ethanol, while no differences were observed for the other cannabinoids (Table 8). As reported by Habschied and coworkers [135], dry hemping also determined a rise in the polyphenol content, which increased from 188 mg L^−1^ in traditional beer to 272 mg L^−1^ in hemp beer; even higher levels (339 mg L^−1^) were reached by adding the dried inflorescences to the wort during boiling (Table 8). Sensory analysis and drinkability test of both hemp beers yielded highly positive feedback, with the highest score and drinkability attributed to the beer added with hemp inflorescences during boiling for its flowery aroma (Table 8).

Ascrizzi and coworkers [133] also produced a hemp-flavored liqueur using the same mix of inflorescences used for producing beer and observed that the liqueur retained more compounds from hemp inflorescences compared to beer (Table 8). Indeed, more than 90% of volatiles in the liqueur were represented by monoterpenes, with α-pinene and myrcene representing more than 50% of the volatile emission. These compounds contributed to the aroma composition of the liqueur, the former with a pine-like and the latter with a sweet aroma. β-Pinene (12.4%), β-caryophyllene (5.8%) and limonene (5.5%) also contributed significantly to the liqueur bouquet (Table 8).

Tea is another popular beverage that can be prepared with herbal blends obtained from dried hemp leaves and inflorescences. Studies on hemp tea have focused primarily on the evaluation of the percentage of cannabinoid release, particularly THC, from the dry material to the infusion to assess its potential impact on consumers. By way of example, Triesch and coworkers [136] observed that the carboxylated forms of cannabinoids transferred to the tea infusion to a much greater extent than the corresponding neutral form (Table 8), which led to an acid to neutral form ratio in hemp tea much higher than in the dry hemp material (on average 68 vs. 2.5 for total cannabinoids). THC concentration ranged between 1.2 and 64 μg L^−1^ of tea; therefore, even at the highest THC concentration, consumption of one cup of tea (200–250 mL) was safe, not exceeding the Acute Reference Dose (ARfD) of 1 μg kg^−1^ of body weight (bw) established by EFSA [137]. Similar findings were reported by Maly and coworkers [138], who analyzed the infusion obtained from the dried inflorescences of two hemp cultivars (Table 8). The results obtained confirmed that the transfer of cannabinoids was higher for the acidic forms compared to the corresponding neutral forms and increased as the polarity of the molecules increased. As for THC, a final concentration of 11–14 μg per single cup of tea (250 mL) was reported, which was far below the ARfD. Similarly to cannabinoids, the transfer of flavonoids was much higher for the highly polar compounds such as vitexin, orientin, and isoquercetin than for the less polar cannflavins A and B (Table 8). Interestingly, the authors observed that the addition of milk cream during the infusion, a practice recommended by some vendors, determined a significant increase in the transfer of both cannabinoids and flavonoids that was greater for less polar compounds. In particular, THC transfer in the presence of cream was such that its concentration increased up to 288–665 μg per cup, which far exceeded the ARfD (e.g., by 4.1–9.5 times for a 70 kg individual) (Table 8).

To date, only one study has been carried out on the use of inflorescences in bread making, in which hemp seed flour is usually most frequently used (see above). Pecyna and coworkers [139] formulated a recipe for a gluten-free bread made with rice flour added with increasing amount (1%, 2%, 3%, 4%, and 5%) of dried and shredded hemp inflorescences (Table 8). This was aimed at improving the quality of gluten-free bread, which is generally characterized by an unsatisfactory texture and taste, as well as a low nutritional value. The results obtained showed that the gluten-free bread added with hemp inflorescences presented an increase in TPC and total flavonoid content (TFC) (from 36% to 195% and from 100% to 433%, respectively) and a consequent increase in TAA (up to 188% and 140% for DPPH and FRAP, respectively) compared to control bread (Table 8). Hemp-infused bread also had an increased volume, reduced hardness and increased chewiness, springiness, and browning of the crust and crumb compared to the control bread. According to consumers, all breads added with hemp inflorescences had a better appearance compared to control bread but, with regard to taste and aroma, they only positively evaluated the samples added with 1% and 2% inflorescences (Table 8).

**Table 8:** Representative studies on food fortification with hemp inflorescences and leaves.

Supplemented Food	Effect on Phytochemicals	Other Effects	Ref.
Beer made by adding fresh hemp inflorescences from cvs. Futura 75 and Uso 31 at the beginning on the threshes, at the end of boiling, and at the end of whirlpooling	Decrease in ethyl hexanoate from 11.7% in control beer to 10.2% in hemp beer, ethyl octanoate from 32.2% to 24.7%, and total non-terpene volatiles from 88.1% to 84.0% Increase in myrcene from 5.6% in the control to 9.8% in the hemp beer, and total monoterpenes from 6.1% to 10.3%.		[133]
Three beers (3%, 6%, and 9% alcohol) made by adding dried hemp inflorescences from cv. Wife during the maturation phase (dry hemping)	Increase in total monoterpenes from 0.42 mg L^−1^ in control beer to 5.36, 6.12, and 6.62 mg L^−1^ in hemp beer with 3%, 6%, and 9% alcohol, respectively Increase in total sesquiterpenes from 0.42 mg L^−1^ in control beer to 1.03, 0.73, and 1.09 mg L^−1^ in hemp beer with 3%, 6%, and 9% alcohol, respectively CBDA at 44.5, 15.3, and 11.45 ng mL^−1^ in hemp beer with 3%, 6%, and 9% alcohol, respectively		[134]
Beer made by adding dried hemp inflorescences at the beginning of the boiling phase or at the end of fermentation (dry hemping)	Increase in the polyphenol content from 188 mg L^−1^ in control beer to 339 and 272 mg L^−1^ in hemp beer with hemp inflorescences added during the boiling phase or after fermentation, respectively	Increase in specific gravity, real extract, original extract, apparent extract, alcohol, and color in hemp beers Higher score and drinkability for hemp beers compared to control beer	[135]
Liqueur made by macerating hemp inflorescences from cvs. Futura 75 and Uso 31 in ethyl alcohol	Volatiles including 38.8% α-pinene, 28.0% myrcene, 12.4% β-pinene, 5.8% β-caryophyllene, 5.5% limonene, 9.5% total sesquiterpenes, and 90.4% total monoterpenes		[133]
Twenty-three teas made by infusion of hemp leaves and/or inflorescences	30.3%, 3.6%, 1.3%, 0.6%, and 20% transfer of CBDA, THCA, CBD, THC, and total cannabinoids, respectively, from dried inflorescences to teas Up to 112,000, 311, 1970, 64, and 126,307 μg L^−1^ CBDA, THCA, CBD, THC, and total cannabinoids, respectively, in teas		[136]
Two hemp teas made by infusion of hemp inflorescences from cv. Tsiza and CBD therapy	Up to 84%, 28%, 4.1%, 1.9%, 99%, 98%, 20%, and 2.4% transfer of CBDA, THCA, CBD, THC, orientin, vitexin, cannflavin B, and cannflavin A, respectively, from dried inflorescences to teas Up to 93%, 99%, 68%, 63%, 99%, 99%, 79%, and 50% transfer of CBDA, THCA, CBD, THC, orientin, vitexin, cannflavin B, and cannflavin A, respectively, from dried inflorescences to teas with cream Up 7366, 33, 893, 14, 338, 106, 16, and 2.9 μg CBDA, THCA, CBD, THC, orientin, vitexin, cannflavin B, and cannflavin A, respectively, per cup (250 mL) of tea Up to 8201, 79, 39,854, 665, 392, 105, 63, and 63 μg CBDA, THCA, CBD, THC, orientin, vitexin, cannflavin B, and cannflavin A, respectively, per cup (250 mL) of tea with cream		[138]
Bread made with rice flour replaced with 1%, 2%, 3%, 4%, and 5% powder of dried hemp inflorescences from cv. Futura 75	Increase in TPC from 0.22 mg GAE g^−1^ in control bread to 0.30, 0.41, 0.44, 0.58, and 0.65 mg GAE g^−1^ in bread added with 1%, 2%, 3%, 4%, and 5% inflorescences, respectively Increase in TFC from 0.03 mg QE g^−1^ in control bread to 0.06, 0.08, 0.10, 0.13, and 0.16 mg QE g^−1^ in bread added with 1%, 2%, 3%, 4%, and 5% inflorescences, respectively Increase in TAA (DPPH) from 1.12 μM TE g^−1^ in control bread to 1.66, 2.08, 2.38, 2.92, and 3.23 μM TE g^−1^ in bread added with 1%, 2%, 3%, 4%, and 5% inflorescences, respectively Increase in TAA (FRAP) from 1.25 μM TE g^−1^ in control bread to 1.25, 1.60, 2.09, 2.27, and 3.00 μM TE g^−1^ in bread added with 1%, 2%, 3%, 4%, and 5% inflorescences, respectively	Increase in bread volume, pH, browning, springiness, and chewiness Decrease in crumb lightness and hardness High acceptability for the appearance of all hemp-infused breads High acceptability for the aroma and texture only for breads infused with 1% and 2% hemp inflorescences	[139]

CBD, cannabidiol; CBDA, cannabidiolic acid; DPPH, 1,1-diphenyl-2-picrylhydrazyl; FRAP, ferric reducing antioxidant power; GAE, gallic acid equivalents; QE, quercetin equivalents; TAA, total antioxidant activity; TE, Trolox equivalent; TFC, total flavonoid content; THC, Δ^9^-tetrahydrocannabinol; THCA, Δ^9^-tetrahydrocannabinolic acid; TPC, total phenolic content.

#### 5.2.2. CBD-Infused Foods and CBD Supplements

CBD-infused foods are widely available on the market and include chocolates, coffee, gummies, candies, baked goods, teas, juices, beers, wines, soft drinks, and liquors, whereas CBD supplements are sold as over-the-counter products in the form of capsules, oils, tinctures, and vapes. The amount of CBD in these products can vary widely depending on the product type and manufacturer, with values ranging from 5 to 30 mg per edible unit, from 20 to 50 mg per capsule, and from 30 to 900 mg mL^−1^ for oils [140]. CBD-based products are used more commonly as a targeted therapy for medical disorders than for overall health and well-being. From surveys carried out in different countries it emerged that the most common symptoms for which consumers appeared to be using these products were pain, stress, anxiety, and depression [141,142,143]. In most cases, consumers stated that the CBD product was effective in alleviating the symptoms of the disease, but a placebo effect cannot be ruled out, especially considering that the doses obtained with the recommended use of such products would be lower than 150 mg day^−1^ CBD, which are far below the effective doses ranging from 300 to 1500 mg day^−1^ established by clinical trials [140]. However, it should be noted that the recommended daily dose may not correspond to the amount actually taken, as these products are often not labeled correctly. In this regard, the Food Standard Agency analyzed 100 samples including CBD edibles and supplements and reported that only 47 samples matched the CBD claim, while 8 and 45 samples had higher and lower CBD levels than claimed, respectively [144]. Also, a study carried out by Bonn-Miller and coworkers [145] on 84 CBD-based supplements sold online revealed that the CBD content was correctly labeled in only 26 products, while it was underlabeled and overlabeled in 36 and 22 products, respectively. In both studies, unlabeled THC was detected in some of the tested samples in amounts high enough to cause intoxication. These inaccuracies in labeling can have negative consequences on the efficacy and safety of products, putting the health of consumers at risk.

## 6. Conclusions

In the present review it was highlighted that hemp phytochemicals represent an attractive source of innovation and opportunity in the agrochemical, cosmetic, and food industry, and that the development and manufacturing of products using hemp ingredients is a very intriguing field of study. In particular, the use of hemp phytochemicals has demonstrated potential in providing advantages for humans and the environment. These bioactive compounds exhibit a wide range of beneficial effects, encompassing antioxidant, anti-inflammatory, analgesic, anxiolytic, neuroprotective, and insecticidal properties, and their incorporation into food and cosmetic products, as well as their use as biopesticides, can amplify their potential utility in improving human health. From an agronomical point of view, hemp cultivation provides several environmental benefits as it plays a crucial role in soil remediation and carbon sequestration and requires little or no usage of chemical pesticides or fertilizers. In addition, the industrial use of hemp leaves, inflorescences and by-products like hemp seed cake or meal, which are all waste of the fiber and seed industry, can help maximize resource efficiency, thus contributing to a more self-sustaining economy and mitigating the environmental impact.

Nonetheless, some aspects need to be carefully considered at every step of the production process. First of all, the choice of cultivar, the use of specific agronomical practices, the choice of the harvesting stage, and the storage conditions are fundamental parameters for maximizing the level of phytochemicals in the various hemp by-products and ensuring supplies of high-quality raw materials that meet industrial needs. Then, rigorous quality control measures must be implemented throughout the entire manufacturing process, which include raw material testing, continuous monitoring during production, and final product testing. This will ensure regulatory compliance, as well as a high standard of the finished product and consumer safety.

Moving forward, further studies are needed to fill the critical gaps that still exist. First, it is necessary to develop cost-effective and scalable extraction and purification methods to produce highly purified hemp phytochemicals while minimizing production costs, energy consumption, and environmental impact. Second, thorough toxicological and safety studies must be carried out to generate reliable data on the long-term effects of hemp phytochemicals in various matrices, including local and systemic toxicity, allergenicity, and interactions with other compounds. Third, validated analytical methods and robust quality control procedures must be developed to standardize the cannabinoid content of commercial products and enable consistent product performance, reliable safety assessments, and greater consumer protection. Addressing these gaps will support the development of reliable, effective, and regulatory-compliant hemp-based products, thus increasing the added value for the hemp by-products. This will help the industrial hemp establish itself as a sustainable and valuable crop, and the benefits of its cultivation will improve the socio-economic status of farmers globally.

## Figures and Tables

**Table 7:** Representative studies on food fortification with hemp seed flour.

Supplemented Food	Effect on Phytochemicals	Other Effects	Ref.
Pasta made with semolina replaced with 5%, 7.5%, and 10% flour from hemp seed cake sieved at 530 mm (Hemp 1_5, Hemp 1_7.5, and Hemp 1_10, respectively) and 236 mm (Hemp 2_5, Hemp 2_7.5, and Hemp 2_10, respectively)	Increase in TPC from 1.11 mg GAE g^−1^ in control to 2.50, 4.25, 4.92, 1.95, 2.76, and 4.21 mg GAE g^−1^ in Hemp 1_5, Hemp 1_7.5, Hemp 1_10, Hemp 2_5, Hemp 2_7.5, and Hemp 2_10, respectively Increase in TAA (DPPH) from 1.14 mmol TE 100 g^−1^ in control to 2.30, 3.08, 3.86, 2.08, 2.65, and 3.14 mmol TE 100 g^−1^ Hemp 1_5, Hemp 1_7.5, Hemp 1_10, Hemp 2_5, Hemp 2_7.5, and Hemp 2_10, respectively	Increase in optimal cooking time, water absorption, adhesiveness, and browning Increase in mono- and polyunsaturated fatty acid, total amino acid, and mineral content High acceptability for Hemp 2_7.5 and low for Hemp 1_10	[127]
Pasta made with wheat flour replaced with 5%, 10%, 15%, and 20% (HSM_5, HSM_10, HSM_15, and HSM_20, respectively) flour from hemp seed cake	Increase in TPC from 13.34 mg GAE g^−1^ in control to 16.12, 16.68, 17.02, and 18.14 mg GAE g^−1^ in HSM_5, HSM_10, HSM_15, and HSM_20, respectively Increase in TAA (DPPH) from 18.48% inhibition in control to 21.57%, 22.06%, 24.16%, and 24.00% inhibition in HSM_5, HSM_10, HSM_15, and HSM_20, respectively	Increase in dough strength and viscosity and decrease in elasticity Increase in browning of pasta during desiccation Increase in optimal cooking time and cooking loss and decrease in water absorption and swelling Increase in protein, fat, and ash content and decrease in carbohydrate content High acceptability for HSM_5 and low for HSM_20	[129]
Bread made with wheat flour replaced with 15%, 30%, and 50% (WH15, WH30, and WH50, respectively) partially defatted hemp seed flour	Increase in TPC from 256.43 mg GAE kg^−1^ in control to 563.63, 604.32, and 673.59 mg GAE kg^−1^ in WH15, WH30, and WH50, respectively Increase in TAA (DPPH) from 294.25 mg TE kg^−1^ in control to 676.36, 772.72, and 895.67 mg TE kg^−1^ in WH15, WH30, and WH50, respectively Increase in total phenolic compounds detected by HPLC from 206.10 mg kg^−1^ in control to 362.96, 566.94, and 754.43 mg kg^−1^ in WH15, WH30, and WH50, respectively	Decrease in bread volume and crumb lightness and increase in crumb browning and hardness Decrease in cohesiveness, gumminess, chewiness and resilience of crumb during storage Increase in protein content High acceptability for WH15 and low for WH50	[130]
Bread made with wheat flour replaced with 5%, 10%, 15%, 20%, 30%, and 40% (HCB5, HCB10, HCB15, HCB20, HCB30, and HCB40, respectively) flour from hemp seed cake	Increase in TPC from 148.25 mg GAE kg^−1^ in control to 182.35, 216.17, 225.84, 256.43, 308.97, and 354.85 mg GAE kg^−1^ in HCB5, HCB10, HCB15, HCB20, HCB30, and HCB40, respectively Increase in TAA (DPPH) from 228.25 mg TE kg^−1^ in control to 318.58, 377.85, 393.86, 452.47, 534.18, and 627.55 mg TE kg^−1^ in HCB5, HCB10, HCB15, HCB20, HCB30, and HCB40, respectively	Increase in baking loss and browning and decrease in volume and porosity Decrease in hardness, gumminess, and chewiness in HCB5 and HCB10 and increase in HCB20, HCB30, and HCB40 Increase in protein and fiber content and decrease in carbohydrate content Good acceptability for all fortified bread	[128]
Bread made with semolina replaced with 5%, 7.5%, and 10% flour from hemp seed cake sieved at 530 mm (Hemp 1_5, Hemp 1_7.5, and Hemp 1_10, respectively) and 236 mm (Hemp 2_5, Hemp 2_7.5, and Hemp 2_10, respectively)	Increase in TPC from 0.54 mg GAE g^−1^ in control to 0.73, 1.22, 1.73, 0.98, 1.11, and 1.64 mg GAE g^−1^ in Hemp 1_5, Hemp 1_7.5, Hemp 1_10, Hemp 2_5, Hemp 2_7.5, and Hemp 2_10, respectively Increase in TAA (DPPH) from 20.20% in control to 28.02%, 38.64%, 46.27%, 22.90%, 34.35%, and 42.08% in Hemp 1_5, Hemp 1_7.5, Hemp 1_10, Hemp 2_5, Hemp 2_7.5, and Hemp 2_10, respectively	Decrease in development time and dough strength Decrease in bread volume and height and increase in crumb and crust browning Increase in α-linolenic and amino acid content Good acceptability for all fortified bread	[131]
Biscuits made with corn flour replaced with 20%, 40%, and 60% (H20, H40, and H60, respectively) hemp seed flour	Increase in TPC from 0.87 mg catechin g^−1^ in control to 1.23, 1.65, and 2.12 mg catechin g^−1^ in H20, H40 and H60, respectively Increase in TAA (ABTS) from 15.10 mmol TE kg^−1^ in control to 22.10, 32.58, and 43.76 mmol TE kg^−1^ in H20, H40 and H60, respectively	Increase in water and oil absorption and swelling Decrease in biscuit volume and increase in biscuit hardness and browning Increase in protein, fat, fiber, and mineral content and decrease in carbohydrate content High acceptability for H20 and low for H40 and H60	[132]

ABTS, 2,2′-azino-bis(3-ethylbenzothiazoline-6-sulfonic acid); DPPH, 1,1-diphenyl-2-picrylhydrazyl; GAE, gallic acid equivalents; TAA, total antioxidant activity; TE, Trolox equivalents; TPC, total phenolic content.

## Data Availability

No new data were created or analyzed in this study. Data sharing is not applicable.
